# Study of Compressibility Properties of Yogurt Powder in Order to Prepare a Complementary Formulation 

**Published:** 2013

**Authors:** Abdolhossein Moghbel, Hamideh Abbaspour

**Affiliations:** a *Department of Pharmaceutics, School of Pharmacy, Ahvaz Jundishapur University of Medical Sciences, Ahvaz, Iran.*; b *Department of Clinical Pharmacy, School of Pharmacy, Shahid Beheshti University of Medial Sciences, Tehran, Iran. *

**Keywords:** Yogurt, Compressibility, Plastic deformation, Tablet processing, Dietary supplements, Lyophilization

## Abstract

The aim of the present study was to prepare an oral tablet from liquid yogurt by reforming the physical properties for easy transportation, long-term storage and also as a complementary dairy product in case of nutrient deficiency. The liquid fresh yogurt was lyophilized at -40°C and 0.03 mTor pressure. The dry powder was homogenized by a 12 mesh size sieve. Some tests such as Carr’s compressibility index, Hausner ratio and the angle of repose were applied to evaluate the flowability of yogurt powder. Study of the deformation of particles during forcing was done by calculation of the elastic recovery index. Carr’s compressibility index percent and Hausner ratio were calculated 15 and 0.94, respectively. The range of repose angle was measured between19-20°. The elastic recovery was obtained up to 60%. Since the hardness of tablets increased by decreasing compression velocity, therefore yogurt powder might have a plastic deformation. The reduction of powder volume due to compression force was about 20% (p < 0.05). Tablets with low fat yogurt showed very good compressibility with 6-12 Strong-Cab (SC) hardness units. Producing a complementary formulated as a tablet from yogurt powder is possible and also maybe therapeutically effective against lactose-intolerance syndrome and preventing antibiotic-associated diarrhea.We suggest that for more authentic confirmation of the type of deformation, it should go through Heckel’s equation analysis, too.

## Introduction

Yogurt is one of the dairy products which is produced by the fermentation of milk with a kind of bacteria. Many researches have been accomplished about the therapeutic and prophylaxis effects of yogurt and the bacteria responsible to produce it in some diseases such as cancers, infections, gastrointestinal disorders and asthma. Yogurt is nutritionally rich in protein, calcium, riboflavin, vitamin B_6_ and vitamin B_12_. Yogurt has nutritional benefits beyond those of milk: people who are moderately lactose-intolerant can enjoy yogurt without ill effects, because the lactose in the milk precursor is converted to lactic acid by the bacterial culture. The reduction of lactose by passes the affected individuals› need to process the milk sugar themselves (1). Yogurt containing live cultures are sometimes used in an attempt to prevent antibiotic-associated diarrhea (2). Yogurt contains varying amounts of fat: non-fat (0%fat), low-fat (usually 2% fat) and plain or whole milk yogurt with 4% fat (3). A study also found that the consumption of low-fat yogurt can promote weight loss, especially due to the calcium in the yogurt (4).”Whey” is a clear liquid which is separated from yogurt. This liquid has many benefits as it has many minerals and is a good source of protein. With respect to nutritious and therapeutic benefits and utilities in human oral basket, it is in a light, solid and portable form, particularly for passengers and mountaineers. For the promotion of yogurt stability in long-term storage, it was decided to make yogurt in a tablet dosage form. The other objective of this study was to invert the liquid phase of yogurt to a solid phase by a proper method for saving nutrient materials, fat and vitamins in a safe medium and far from destructive temperature.Therefore, in the first step it is necessary to put to the test whether the yogurt powder could be compactable or not. For this purpose and taking into consideration the lab possibilities,the following concepts and technological understanding should be considered. Compactibility is the ability of a powder bed to cohere into or to form a compact with reduced volume and specified tensile strength (5-7). During compression, increasing the force leads to particle deformation and rearrangement. At this point, the three principal modes of deformation are as follows: elastic deformation, plastic deformation and brittle fracture. The phenomenon named plastic deformation in a powder form during exerting a force is an irreversible occurrence which results in producing stronger tablets. Whereas, elastic deformation is a reversible phenomenon and at a decompression phase when the force is released the connections between particles undergo brittle fracture if the shear strength between the particles is greater than the tensile or breaking strength. Under these conditions, larger particles are sheared and broken into smaller particles and capping may occur (8, 9). Microcrystalline cellulose (MCC), pre-gelatinised starch and sodium chloride have plastic deformation during compression and Emocompress^®^ (calcium phosphate dihydrate), crystalline lactose, paracetamol, and ascorbic acid are compounds that undergo a fragmentation stage (10-12). The mechanism and principle stages of binding interfere to produce a compressed solid form are as follows: formation of a solid bridge by strong forces between particles, long distance intermolecular forces such as Vander Waals, electrostatic or hydrogen bonding and mechanical links which are established on husking or twisting by chance to form new particles. The most important bonding mechanism occurring in drug powders are long distance forces. For example in sodium chloride Vander Waals and hydrogen binding are prerequisite to be formed the solid bridges needed to make a plastic deformation (13, 14).

Usually, tensile strength has an inverse relation to the particle size (15,16) but if the particles were broken due to a compression force, in that case, the particle size and shape have less effect on the strength of tablets (17). Factors which help to separate the upper, lower or intermediate layers of tablets (18) are as follows: low compressibility, weak bonding and long flexibility or elasticity of the powder. In such situations, the granulation of the powder may improve the difficulties (19). It is reported that multiple compaction might result in changing of compressibility properties and usually because of decreasing in bonding potential, less tensile strength is observed (20, 21). A method which is applied for a volume decrease of a powder during compressibility process is called Heckel equation and porosity-pressure function (curve). This equation assumes that the volume decrease process of particle pores during compressibility follows first order kinetics (22). Parameters affecting compressibility properties may be obtained by drawing porosity-pressure curve. Yield to pressure (YP) is used for plasticity measurement and it is comparable criterion for compressibility observation. Heckel equation is mostly used for plain material, but it may be used for mixed powder with precautious (23). Also, Heckel equation could be used for dry granulation (24). Some limitations for using Heckele quation analysis about the difference between the density of the material and also small laboratory errors are reported (25-28). Heckele quation is affected by particle size, too (29). Wet granulation is influenced by rearrangement and densification of initial powder, and if so, the Heckel equation could describe the results (30). Fragmentation phenomenon may be evaluated via sensitivity to lubricant, indirectly. For plastic material,the lubricant could decrease intermolecular forces and also surface free energy, therefore resultingin the reduction of the tensile strength. Whereas, the lubricant has no any influence on fragmented material (10, 31). Volume reduction could also be observed by scanning electron microscope (10, 12, 32). In this study there is an attempt to produce a tablet from yogurt for complementary or therapeutic uses. Therefore, the first step was to evaluate the properties of the prepared powder from yogurt such as compressibility, flow ability and deformation behaviour.

## Experimental

*Preparation of yogurt lyophilized powder*

A quantity of 2 kilograms of a fresh semisolid native yogurt was filtered and then lyophilized at -40°C and 0.03 mTor pressure by a freeze dryer (ZirbusZa co-5, Germany) for about 8 h (5). The lyophilized powder was homogenized by a 12 mesh sizedsieve.

*Flowability evaluation of yogurt powder*

*Carr’s compressibility index*

This test would evaluate the relationship between flowability and compressibility of a powder (6). A quantity of 100g of lyophilized yogurt powder was filled into a graduated glass cylinder and repeatedly tapped on a shaker. The sample was tapped for 500 times and repeated at 3 turns. The volume of powder after tapping and Carr’s index percent was measured as follows: 

$\mathrm{Carr's index}(\%)=\frac{\mathrm{Poured or bulk density}}{\mathrm{Tapped density}}\times \mathrm{100}$                    (Equation 1)

$\mathrm{Bulk density}=\frac{\mathrm{Wiegh}}{\mathrm{Bulk Volume}}$                    (Equation 2)

$\mathrm{Tapped density}=\frac{\mathrm{Wiegh}}{\mathrm{True Volume}}$                      (Equation 3)

The relationship between powder flowability and % compressibility are shown in Table 1 (6, 7).

**Table 1:** Relationship between powder flowability and % compressibility

**Flow description**	**%Compressibility**
Excellent Flow	5-15
Good	16-18
Fair	19-21
Poor	22-35
Very poor	36-40
Extremely poor	>40

*Hausner ratio*

This test is related to inter-particle friction and is defined as follows (6-8): 

Hausner ratio =$\frac{\mathrm{Tapped density}}{\mathrm{poured or bulk density}}$

 (Equation 4)

In this test, values less than 1.25 indicate good flow (≈ 20% Carr), a value greater than 1.5 indicate poor flow (≈ 33% Carr).

*The range of repose angle (*ϕ)

In this test the sample is poured on to a horizontal surface and the angle of the resulting pyramid is measured. The frictional forces in a loose powder can be measured by the angle of repose (ϕ) which is the maximum angle possible between the surface of a pile of powder and horizontal plane which is equal to the coefficient of friction μ between the particles and is mathematically shown as follows (33): 

Tan ϕ *= *μ                     (Equation 5)

Tan ϕ =$\frac{h}{r}$                      (Equation 6)

r =$\frac{d}{2}$                     (Equation 7)

Interpretation of the result is shown as classified in Table 2. 

**Table 2:** Interpretation of the angle of repose

**Flow description**	**Repose angle (**o**)**
Excellent	Less than 20
Good	20-30
Pass	30-34
Poor	Greater than 40

Also, the rougher and more irregular surface of the particles could result in the higher angle of repose. This test was done on 200g of lyophilized powder and at the condition of 25 °C and 50-60% relative humidity (7, 9).

*Study of yogurt lyophilized powder compressibility*

*Deformation evaluation*

The compactibility of a powder mostly depends on two factors, first granulation and second, deformation of particles during forcing. Deformationin the particles of a powder under forcing may happen in the form of so-called: plastic, elastic or fragmentation.

*Applying the elastic recovery index*

The elastic recovery was evaluated by the following equation: 

$\mathrm{ER}=\left[\frac{H_{p}-H_{p}}{H_{p}}\right]\times 100$                     (Equation 8)

*H*_p_= Tablet thickness after compression

*H*_0_= Tablet thickness after release of compression force 

In this test after tablet ejection, the elasticity was evaluated, using 500 mg yogurt powder in a 10mm die and applying the punches force to be compressed. The thicknesses of the compressed tablets were measured immediately after ejection by a tablet thickness tester (Vernier Caliper, ENGLAND). For calculating the exact ER value the true density of the powder (the density at maximum pressure and zero porosity) was needed but because of nitrogen defect of the instrument, the tapped density was substiluded. Therefore, using the following equations the volume and finally the elastic recovery was calculated.

$\rho \mathrm{tap}=\frac{m}{v}$                     (Equation 9)

*V*= 3.14R^2^H                    (Equation 10)

Also, in the subject of deformation of a powder involved in a force for compressibility, the rate of compactibility is needed to be evaluated on the hardness of the powder.

*Evaluation of the rate of compactibility on the hardness of yogurt powder*

In this test, the hardness change was occuring at two different compression rates of slow (with the lag time) and quick range.

*Lubricant sensitivity test*

In this test if the hardness of a compressed tablet decreased with the increase of lubricant the powder assumes to be plastic but, if it doesn’t make any change, it is said to be a fragment powder. In practice, 500 mg of yogurt powder was mixed for 10 min with 1% magnesium stearate and once without the lubricant and then both samples were compressed. From each sample 10 tablets were picked up randomly at 1 h after compression for testing of the hardness by the Strong–Cab hardness tester. Each test was repeated three times.

*Evaluation of deformation type with respect to the time of mixing and dwell time*

A quantity of 500 mg yogurt powder was mixed with 1% magnesium stearate and was compressed at a constant compression force of 75MP with respect to the condition mentioned in Table 3. Hardness values were used to determine deformation. The punches and matrices were constant in all formulations.

**Table 3:** Variables of mixing time and duration of compression (dwell time).

**Variables**	**Formulations**		
	A	B	C
Mixing time (min)	5	5	30
Dwell time (Sec)	2	30	2
Hardness after 24hrs (N)	*AN*	*B*_N_	*C*_N_

*Evaluation of the yogurt fat lubrication effect*

In this study two different formulations of yogurt powder were prepared. The first formulation contained 2% NaCl and the second one 2% dibasic calcium phosphate, besides 2% sodium chloride.

*Granulation and particle rearrangement*

Granulation is one of the most important ways for the fine particles to increase the size distribution, homogenizing and making spherical the shape of particles and finally increasing the tendency of compressibility in rough particles. In this test, four types of yogurt granules were evaluated as follows:high fatty (3%), low fatty (1.5%) filtrated whey; low fatty infiltrated and a sample of very homogenized infiltrated low fat yogurt. 

## Results

*Deformation type*

The elastic recovery was calculated 60% using equations No 8-10. The hardness of tablets increased by decreasing compression velocity.This means that the yogurt powder might have a plastic deformation.

*Lubricant sensitivity result*

The sensitivity to lubricant test showed a reduction in the hardness of tablets. Also, magnesium stearate which was used as a lubricant caused a significant decrease (p < 0.05) in the hardness of tablets (Table 4).

**Table 4:** Lubricant effect on the hardness (Strong-Cab) of yogurt tablets

**Hardness with lubricant**	**Lubricant free**
2.5	6
3	5.5
2	6
2.5	6.5
3	5
3.5	6.5

*The mixing and dwell time result*

Evaluation results of comparable parameters: *A*_N_*, B*_N_ and *C*_N_ on the Table 1 were as follows: *B*_N_> *A*_N_* > C*_N_ These arrangements meant that the hardness of the condition B (less mixing time with higher dwell time value, was larger than condition A (lower both mixing and dwell time), and both of them were larger than condition C (higher mixing and lower dwell time), in comparison.

*Fat lubrication effect*

The result of this study showed that adding of dibasic calcium phosphate caused an increasing trend in hardness from 2 to 5 (SC)

*Relation of volume-compression force*

The reduction of yogurt powder volume due to compression force was about 20% (p < 0.05).

*Granulation results*

*Compressibility and hardness*

The yogurt granules prepared from high fat yogurt had not enough compressibility to produce normal tablets. Tablets were brittle with very low adhesiveness and hardness. Tablets prepared from low fat yogurt showed very good compressibility with 7-13 SC hardness. Tablets prepared from granules with low fat (1.5%) and whey filtrated yogurt had no good hardness even with changing compression force of the tablet machine.

*Homogeneous effect*

The granules and tablets prepared from low fat and whey infiltrated yogurt which went through with the homogenizing process before drying and granulation showed a good homogeneous on surface of tablets with a better compressibility on the granules and the hardness of tablets.

## Discussion

Since tablets have an elastic recovery of about 2-10% (34), some of the devices should be adjusted to reduce this factor, as much as it needed to be desirable. However, on the other side, having a high elastic recovery could be a reason of plasticity for total yogurt. Anyhow, it needs to be investigated further. Since the hardness of yogurt tablets increased by the compression velocity, therefore, the hardness of yogurt tablets is related to time and it results due to above reason that the yogurt powder might have a plastic property. The decrease for hardness in the presence of the lubricant could be another reason of plasticity of yogurt powder. Additionally, evaluation of mixing and dwell time (duration of applying force) which showed an increase in hardness by increasing in dwell time and decreasing in the mixing time, are probably other signs of plasticity. Anticipation of the lubricating effect of the yogurt’s fat showed that the addition of dibasic calcium phosphate increased the hardness from 2 SC to 5 SC.This defined that yogurt’s fat probably exerted a lubricant role in the yogurt powder and this effect could be a good sign of plasticity of the powder. Incompactibility phenomenon;two factors such as deformation and particle rearrangement are important in producing a tablet with desired hardness (25, 27). Therefore, it seems that the problem of no compressibility or low compressibility of the complete yogurt powder is because of particle rearrangement which could improve by granulation process. In tablets which were produced by the granules of complete yogurt with no separation of the water and 1.5% fat, the hardness of tablets could increase as much as 7-13 SC. This result showed the specific effect of yogurt’s water (whey) on the hardness of yogurt tablets.In order to study deformation more accurately and also specifically, the Heckel equation (17, 18) should be applied. For this study true density is needed which could be obtained by using a gas pycnometer. Because of nitrogen deficiency of the instrument, it was not possible to complete the test in our lab but, the process could be done as follows: after measuring the thickness of prepared tablets (mean of ten) by a thickness tester the apparent density would be calculated by dividing the weight (mass) by apparent volume of the powder. Finally,the relative density (D) could be achieved by dividing the apparent density by true density, which is practically obtained by a gas pycnometer. 

After that, applying equation No.11 and drawing the graph of *Ln *(1/1-D) versus compression force (p), according to this formula the linear part of the curve would have the slope of K which is called a yield value. This parameter may be used as a quantitative standard for determination of the plasticity property of yogurt powder. 

(Ln 11-D=KP+A)                     (Equation 11)

**Figure 1:**
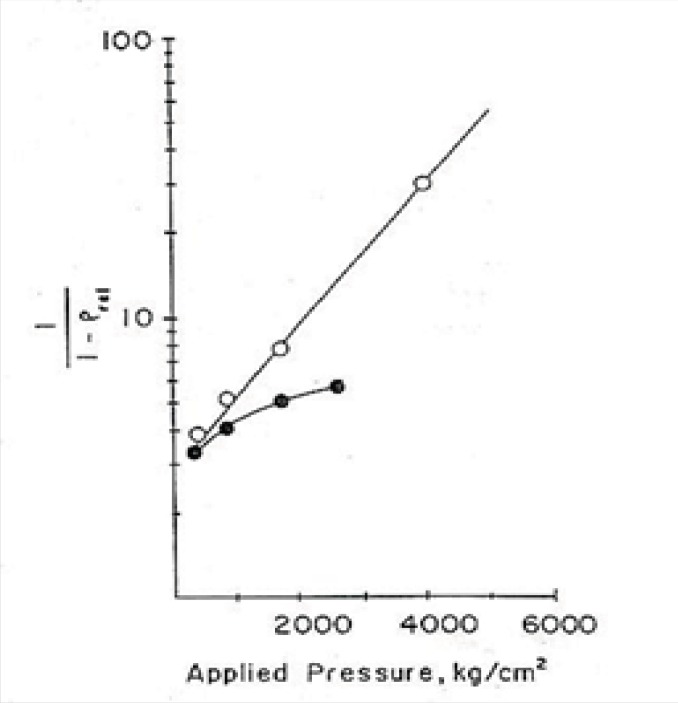
Density-applied pressure relationship according to the Heckel plot:●,dibasic calcium phosphate dihydrate;○,with 4.5% starch (22).

In Figure 1, the plasticity behaviour of dibasic calcium phosphate dihydrate and with 4.5% starchare shown, for example (22).

## Conclusion

Since, the hardness of prepared tables of yogurt powder increase by decreasing in compression velocity, thus it might have a plastic deformation type. The tablets prepared with unfiltered (containing whey) low fat yogurt had a good compressibility during compression.
